# Brain sustain partnerships: Equity-orientation and decolonization theory to promote Global North-Global South collaborations in health professions education

**DOI:** 10.1371/journal.pgph.0007630

**Published:** 2026-11-11

**Authors:** Robin K. Enns, Maria Athina Martimianakis, Elizabeth Peter, Freida Chavez, Denise Gastaldo

**Affiliations:** 1 School of Nursing, Faculty of Health Sciences, McMaster University, Hamilton, Ontario, Canada; 2 Bloomberg Faculty of Nursing, University of Toronto, Toronto, Ontario, Canada; 3 Department of Paediatrics, Wilson Centre for Research in Education, Temerty Faculty of Medicine, University of Toronto, Toronto, Ontario, Canada; 4 Joint Centre for Bioethics, University of Toronto, Toronto, Ontario, Canada; 5 Dalla Lana School of Public Health, University of Toronto, Toronto, Ontario, Canada; 6 Department of Family and Community Medicine, Temerty Faculty of Medicine, University of Toronto, Toronto, Ontario, Canada; Islamic Azad University South Tehran Branch, IRAN, ISLAMIC REPUBLIC OF

## Abstract

Academic partnerships between institutions in the Global North (GN) and Global South (GS) are promoted as a strategy to strengthen health systems and build workforce capacity, particularly through health professions education. At the same time, growing calls to decolonize global health partnerships have highlighted persistent inequities in power, knowledge production, and resource distribution that shape how these are enacted. While existing literature often emphasizes technical and outcome-oriented models of collaboration, the relational and processual dynamics that sustain or constrain partnerships in practice have been significantly under-examined. This qualitative study is a postcolonial exploration of academic global health partnerships and how they are experienced and negotiated by those involved. Nineteen participants with experience in long-term partnerships between GN and GS universities were interviewed using semi-structured interviews. Data were analyzed iteratively and thematically, revealing how people, processes, and power shape partnership work. Participants described partnerships as fundamentally relational, sustained by individuals who invested significant emotional and relational effort over time. Partnership processes were iterative and required ongoing negotiation of roles, expectations, and priorities in response to changing contexts. Power operated across relationships and processes, shaping whose expertise was recognized, how decisions were made, and how collaboration unfolded. Participants also described moments in which power was acknowledged, reflected upon, and, at times, resisted in efforts to create more egalitarian partnerships by mitigating systemic colonial practices. These findings suggest that academic global health partnerships are not sustained primarily through formal structures or predefined outcomes, but through the dynamic interplay of people, processes, and power. We introduce the concept of “brain sustain” to reframe health workforce development as a collective, relational, and contextually grounded endeavour. Advancing decolonial and equitable partnerships requires recognition of global, historical, and structural conditions, alongside promotion of the relational and processual work that sustain collaboration.

## Introduction

Academic global health partnerships are increasingly operating within a context of geopolitical instability and widening global inequities. Recent shifts in political leadership, the erosion of multilateral commitments, and disruptions to longstanding global health infrastructures have raised concerns about the sustainability of health systems in the Global South (GS), while the Global North (GN) continues recruiting GS health professionals rather than relying solely on educating its own workforce. Globally, there is a severe shortage of health workers, which is exacerbated by their inequitable distribution. Health workers exemplify the ‘inverse care law’, whereby areas with greatest health needs have fewest resources. This can be seen both between and within countries [1].

In this context, GN-GS partnerships that strengthen health professions education and workforce capacity can be seen as essential building blocks of sustainable health systems or as facilitators for brain drain [1]. “Brain drain” is defined as the migration of health personnel seeking a better standard of living, working conditions, technologies, higher wages, and favorable political contexts [2]. This raises questions about whether efforts to strengthen training capacity in the GS divorced from a vision for the sustainability of national healthcare systems may inadvertently contribute to greater inequities between the GN and the GS [3].

Drawing on the experiences of global health partners engaged in long-standing collaborations, this study shows that meaningful and sustainable partnerships are not achieved through formal agreements, meeting targets, or traditional outcomes. Instead, they are supported through relational labour, enacted through negotiated processes, that are also shaped by enduring power asymmetries which point towards the need for critically reflexive and equity-oriented approaches. We, therefore, introduce the concept of “brain sustain” as an aspirational counterpoint to brain drain. We define brain sustain as the strengthening and long-term retention of health workforce capacity through the creation of equitable educational, professional, and systemic conditions that enable health workers to develop expertise, generate and mobilize locally relevant knowledge, build meaningful careers, contribute to their communities, and thrive within their national health systems. In contrast to brain drain, which foregrounds the migration of health workers away from the systems in which they were trained, brain sustain centres the conditions that enable health workers to remain, contribute, and flourish within their national health systems.

Importantly, brain sustain is conceptualized as both an aspirational ethos and a practical orientation for academic global health partnerships. It is grounded in strategies developed by study participants, including the work of partnership champions who draw on locally responsive approaches to navigate substantial structural constraints demonstrating commitment, persistence, and other moral and epistemic values. Although individual partnerships cannot independently alter the broader geopolitical, economic, or colonial structures that shape health worker mobility and health system sustainability, partners have meaningful agency within these constraints to enact strategies that support more sustainable forms of partnership for health workers to create conditions for health workers to build careers, contribute meaningfully, receive recognition, and make advances within their national health systems. Seeking to strengthen these conditions and create anti-colonial global partnerships are often perceived as lofty aspirational goals that are difficult to achieve. This research challenges this perception by utilizing the concept of brain sustain which offers an equity-oriented alternative for partnerships and promoting a national healthcare ethos rather than exporting and reproducing GN standards and norms.

Academic partnerships in global health have become a prominent strategy for addressing inequities in health systems and workforce capacity, particularly through collaborations in health professions education between institutions in the GN and GS [4–7]. These partnerships take multiple forms, including capacity-building initiatives, curriculum co-development and delivery, faculty exchanges, and long-term institutional collaborations, and are often described as mechanisms for strengthening educational infrastructure, expanding faculty capacity, and supporting locally relevant training programs for current and future health professionals [8,9]. In the context of persistent global health workforce shortages, such collaborations continue to be positioned as contributing to health systems strengthening and population health promotion. However, this framing rests on an assumption that partnerships function primarily as vehicles for individual and group capacity-building, with less attention to how they are shaped by broader geopolitical shifts and structural inequities. Recent changes in global health governance – including reductions in multilateral engagement and disruptions to longstanding funding and institutional arrangements – have introduced new uncertainties for partnerships and the health systems they aim to support [10–13]. At the same time, efforts to expand training capacity are complicated by ongoing challenges such as workforce migration, raising questions about whether partnerships designed to strengthen local systems may inadvertently contribute to uneven global distribution of health professionals.

Partnerships matter because they are not merely symbolic expressions of solidarity; they actively shape access to resources, opportunities for education and research, institutional priorities, and the distribution of benefits within global health work. Decisions about curricula, funding flows, research agendas, authorship, and mobility materially influence whose knowledge is legitimized and whose priorities are centered [14,15]. As such, academic partnerships are social and political practices that can either contribute to more equitable forms of collaboration or reproduce existing global inequities. Calls for reform emphasize the need for collaborations rooted in respect, honesty, equity, mutual capacity building, and alignment with GS-defined health priorities [16–18]. Pragmatic guidance has also emerged to support more equitable academic partnerships, including attention to governance, reciprocity, transparency, and shared ownership among partners [4,7,17]. In global health education specifically, scholars have called for rethinking institutional partnerships and educational approaches as part of broader decolonizing efforts [18–21].

Despite these commitments, a growing body of scholarship documents persistent inequities within GN-GS academic partnerships [16,20,22]. These include patterns of brain drain, extractive research practices, and asymmetric funding and agenda-setting that privilege GN institutions. Power imbalances are embedded across funding opportunities, intellectual property rights of curriculum, knowledge production and transfer, and access to training resources, shaping whose contributions are rendered visible and whose labour is undervalued [17]. Empirical analyses of academic partnerships further highlight how institutional hierarchies, differential access to resources, and uneven influence over decision-making create both possibilities and pitfalls for collaboration, even within equity-oriented partnerships [4,7]. Historically, many partnerships have been embedded within broader GN-GS political and economic relations shaped by colonial rule and its contemporary afterlives, contributing to ongoing colonial continuities in global health practice and research.

Calls to decolonize global health have intensified in recent years, emphasizing the need to confront epistemic injustice and structural inequities embedded within global health institutions and knowledge systems [15,16,23–26]. While there is broad agreement among global health practitioners that current practices and policies require reform, there is considerable divergence in how decolonization is understood and operationalized and progress in moving from rhetoric to reform remains complex [27,28]. Equitable partnerships require sustained attention to structural determinants and power asymmetries; without such attention, well-intentioned collaborations risk reinforcing the inequities they seek to address [15,16].

Despite extensive documentation of inequities and calls for reform, there remains limited empirical attention to how academic global health partnerships between GS and GN are enacted in practice, particularly with respect to how relational dynamics, partnership processes, and power relations are negotiated in everyday collaboration. In addition, while concepts such as brain drain have shaped discussions of workforce sustainability, often highlighting the loss and maldistribution of health professionals across contexts [2,29–31], there remains limited conceptual work that reimagines sustainability of these partnerships in ways that centre relationality, sovereignty, and locally grounded capacity.

Therefore, the purpose of this qualitative study was to critically examine long-standing academic global health partnerships in health professions education involving partners from the GN and GS. Specifically, we aimed to: (1) explore how partners experience and navigate the relational and procedural dimensions of partnership work, and (2) examine how power relations shaped by historical and contemporary inequities are negotiated in everyday practice. Postcolonial theory was used as a theoretical framework because it offers a lens for naming and interrogating these dynamics by foregrounding how historical and contemporary power relations shape partnership practices, knowledge production, and institutional arrangements.

## Methodology

### Ethics statement

Ethical approval was obtained from the University of Toronto Research Ethics Board. Participants provided verbal and written informed consent prior to participation and were assured of confidentiality, voluntary participation, and the right to withdraw at any time. Given the inherent power asymmetries between GN and GS-based participants, ethical considerations extended beyond procedural ethics to situational and relational ethics [49]. During interviews, participants were viewed as experts on their own partnership experiences, and the use of storytelling allowed participants to frame what mattered most to them rather than responding solely to researcher-driven prompts. Care was taken to avoid deficit-oriented questioning and to explicitly invite accounts of strengths, successes, and challenges. Participants were given pseudonyms to protect confidentiality.

### Theoretical framework

Postcolonial theory provides a critical lens for examining how historical and ongoing colonial relations shape global health, international partnerships, and knowledge production. It centres issues rooted in colonial histories and their aftermath, drawing attention to how people’s lives and life opportunities continue to be shaped by colonial relations [32–35]. Postcolonial theory seeks to uncover, challenge, and critique dominant hegemonic discourses – particularly those related to power – that are intrinsic to relationships between countries in the GN and GS [36,37]. Although colonialism as formal rule has ended, neocolonial economic structures, epistemic dominance, and unequal power relations persist in both the practice and discourse of global health [15,24]. For example, colonial legacies continue to shape who defines problems, whose voices are heard, and which forms of knowledge are valued, rendering power relations not incidental but foundational to how global health problems are defined, researched, and addressed [16]. Anchoring a study of academic global health partnerships between GN and GS countries in postcolonial theory was therefore pivotal for understanding how these partnerships may both reproduce and resist hegemonic power structures and entrenched global inequities.

Two interrelated concepts derived from postcolonial theory were particularly meaningful for this study: decolonizing knowledge production and a spirit of appreciation. Decolonizing knowledge production involves actively challenging the privileging of Western forms of knowledge and creating space for diverse, contextually grounded ways of knowing [19,38]. It requires questioning whose knowledge counts, who has the authority to interpret data, and how research questions are framed and legitimized [37]. In the context of global health, this approach seeks epistemic justice by ensuring that marginalized voices are not only included, but meaningfully involved in shaping the standards of evidence, interpretation, and meaning [15]. Decolonizing knowledge production thus calls for a redistribution of epistemic authority and an ongoing critical examination of the norms and practices that sustain knowledge hierarchies.

The second concept, a spirit of appreciation, foregrounds respect for participants’ expertise, lived experiences, values, and contexts, rather than evaluating partnerships solely through externally imposed benchmarks of ‘progress’ or ‘quality’ [39]. This perspective allows for the exploration of partnerships’ successes, strengths, and resilience while simultaneously acknowledging their challenges and tensions. Together, these concepts sensitized the analysis to relational practices (people), evolving partnership work (processes), and the operation of power across levels (power).

### Study design

This study employed a generic qualitative research (GQR) design, which allows for methodological flexibility while maintaining epistemological coherence [40]. GQR enabled the use of analytic tools drawn from established qualitative methodologies while aligning with the study’s exploratory aims and critical epistemological stance [41]. Anchoring the study in postcolonial theory enabled critical attention to power relations, historical contexts, and the persistence of colonial logics within global health partnerships. The integration of postcolonial theory with a GQR design allowed for epistemological congruence by both enacting and demonstrating critical commitments throughout the study design, data generation, and analysis [42].

### Recruitment and sampling

Participants were recruited using purposeful and snowball sampling strategies. Eligible participants were individuals actively involved in multiple stages of an international partnership in health professions education between at least one GN country and one GS country. Inclusion criteria required participants to have been involved in partnerships that: (1) were university-to-university collaborations; (2) were long-term or ongoing (defined as three or more years); and (3) focused on strengthening or scaling up health professions education through curriculum development, delivery, and/or faculty development in the GS country. Partnerships focused solely on international service-learning clinical placements for learners from GN or humanitarian relief efforts were excluded. Participants from a wide range of GS and GN countries were contacted by email, provided with study information, and invited to participate in an interview by first author, RKE. Participant recruitment occurred between 01/02/2020 and 15/05/2022. Recruitment commenced immediately prior to the COVID-19 pandemic; however, pandemic-related disruptions substantially limited recruitment during the early months of the study. Recruitment activities were therefore temporarily paused and resumed 01/09/2020, continuing until sufficient information power was achieved.

### Data generation

Data were generated through semi-structured interviews conducted in English and recorded via Zoom. Interviews were conducted between 16/10/2020 and 07/06/2022. Conducting interviews over this period enabled participation from collaborators across multiple countries while accommodating evolving pandemic-related constraints and participants’ professional responsibilities. Nineteen participants were interviewed by RKE using a semi-structured interview guide informed by qualitative interviewing principles [43,44] and Smith’s [45] decolonizing principle of storytelling. Storytelling was intentionally used as both a method and a meaning-making process, allowing participants to share diverse truths situated within specific social, political, and historical contexts. Participants were invited to recount stories of both successes and challenges within partnerships, reflecting the study’s commitment to a spirit of appreciation and recognition of complexity [46,47]. In the interview guide (see S1 Appendix), we explicitly explored power relations within partnerships. Participants were asked about the power dynamics that shaped their partnership and how power relations were discussed among partnership members, with probes addressing agenda setting and decision-making. Additional questions about power explored shared leadership, equitable benefits, and participants’ perspectives on an ideal partnership. Interviews were transcribed verbatim and de-identified prior to analysis and participants were given pseudonyms in knowledge translation products including this manuscript. In addition to analytic depth, sufficient information power [48] was assessed in relation to multivocal representation [49], particularly the inclusion of GS participant perspectives that are frequently and historically under-represented. Attention was paid to ensuring that diverse positionalities and partnership roles were represented with sufficient depth to allow for critical and contextualized interpretation [45].

### Data analysis

Data analysis was managed using NVivo software and followed an iterative, three-stage process incorporating inductive, deductive, and abductive reasoning [50–53]. In the first stage, an inductive approach was used to generate descriptive findings related to partnership processes, relationships, and experiences. Thematic analysis was utilized and was guided by Creswell’s analytic steps: organizing data, reading and memoing, coding, generating themes, representing findings, and interpreting meaning [50]. Coding was conducted iteratively, with codes and themes refined through frequent comparison across transcripts. Attention was paid to both convergence and divergence across participant accounts to ensure multivocal representation. In the second stage, deductive analysis was guided by postcolonial theory to examine power relations, marginalization, and epistemic dynamics within partnerships. Abductive reasoning supported movement between empirical data and theory, enabling the refinement of theoretical interpretations as analysis progressed. Stages 1 and 2 were conducted from 07/06/2022 until 31/03/2023 as part of the first author’s PhD dissertation [54].

In the third stage, we undertook a secondary data analysis to consider how meanings were affected by the recent changes and current context of academic global health partnerships. This analysis was conducted between 01/09/2025 and 30/04/2026 and built upon the previous inductive and deductive analyses. The secondary analysis involved returning to the original transcripts, codes, and themes and examining the data with particular attention to the implications of changing geopolitical and funding contexts for global health partnerships. While the post-2024 developments were not part of the empirical dataset, they informed our interpretation of the existing data and our consideration of how lessons from participants’ experiences might inform alternative approaches to partnership in the current context. This iterative re-engagement with the data contributed to the development of the concept of brain sustain, which we propose as an explicitly decolonizing orientation for sustaining health professions capacity and partnership in the face of contemporary challenges to global health.

Postcolonial theory functioned as a sensitizing and interpretive framework rather than a prescriptive coding structure. Theoretical principles informed analytic decisions, particularly in identifying how power, privilege, and historical legacies shaped partnership dynamics and knowledge production. This approach supported theoretically informed findings while remaining grounded in participants’ narratives.

Coding and theming were conducted initially by RKE and later included consultation with authors DG, MAM, EP, and FC to refine interpretations, challenge analytic assumptions, and enhance methodological rigour. Rigour and quality were further enhanced using criteria outlined by Caelli et al. [41] and Tracy [49]. Strategies included maintaining an audit trail, writing analytic memos and reflexive journal entries throughout data generation and analysis, attending to study coherence, and intentionally incorporating multivocal perspectives (particularly those of GS participants, who are often under-represented in the literature) [49]. Reflexive practices were embedded throughout the study as both a methodological and ethical strategy [55].

### Reflexivity

Reflexivity was central to the study’s critical qualitative approach. RKE, a white, upper-middle-class cis woman born and residing in Canada, critically examined how her multiple identities, institutional privilege, positioning within a GN country, and her own experience teaching in GS countries within academic global health partnerships shaped the research process. The tension between being brought up in the GN and having spent time working with academic global health partnership in the GS allowed her to propose a study to explore the discrepancies between differently resourced settings, the complexity in partnerships, and to question the power dynamics implicit in these partnerships as a result of neocolonial globalization.

As a team, our reflexive engagement throughout the study supported efforts to challenge taken-for-granted assumptions and to attend to power relations inherent in global health research. Either our individual migratory experience or international scholarship helped us to experience social relations from multiple locations and problematize the experiences of GN and GS participants. In this way, reflexivity functioned as both a decolonizing strategy and a practice of rigour [45,49].

## Results

Nineteen participants (10 from GN countries and 9 from GS countries) representing 13 long-term academic global health partnerships were included. The partnerships involved institutions in the GN from North America and Europe working with partner institutions or organizations in the GS from Central America, Southeast Asia, and Sub-Saharan Africa. They varied in duration, size, scope, and institutional configuration, including university-to-university collaborations and partnerships involving government agencies, non-governmental organizations, and other educational institutions. Health professions represented included undergraduate medicine and medicine specialities as well as nursing and midwifery, with partnership activities spanning curriculum development and implementation, faculty, specialty training, strengthening health systems and program evaluation, and improving population health. Partnerships ranged from recent collaborations (five years or less) to collaborations extending over two decades. All participants had extensive experience working in global health partnerships and occupied a range of professional roles within these collaborations and many participants articulated explicit commitments to equity, social justice, and, in some cases, decolonizing approaches to their global health work. This context is important for interpreting the findings, as it reflects partnerships that had already engaged critically with questions of collaboration, power, and sustainability. Further details regarding the partnerships presented in the study are available in the first author’s doctoral dissertation [54].

Findings are organized around three interrelated domains of academic global health partnerships: people, processes, and power. By centering relational labour, processual negotiations, and the operation of power dynamics, this study reveals recurring tensions and possibilities for reorienting partnerships towards equity-oriented and decolonial practices in global health education and research. The sections that follow explore how participants described partnerships as fundamentally relational, enacted through evolving and negotiated processes, and shaped by power operating across interpersonal, institutional, and structural levels. This perspective differs from the traditional, often technically oriented models of partnership [56].

### People: Relational labour and commitment

Participants consistently described academic global health partnerships as fundamentally relational endeavours. Relationships between partners were not peripheral to collaboration but constituted the primary mechanism through which partnerships were formed, maintained, and navigated through uncertainty. Central to these relationships were two interrelated features: the involvement of partnership champions who invested deeply in the relational work of sustaining partnerships over time and a set of shared anticolonial values that oriented how partners viewed and enacted their partnership work. Together, these elements highlight that partnerships were not maintained through structures or formal agreements alone, but through ongoing relational labour and a shared ethical commitment to how collaboration should unfold.

### Champions and the labour of sustaining partnerships

Across interviews, the importance of champions – individuals who invested significant emotional, relational, and administrative labour to sustain partnerships over time and were deeply committed to the partnership – became apparent. These individuals played critical roles in initiating collaboration, maintaining momentum, and navigating challenges, particularly during periods of instability such as funding interruptions, institutional restructuring, or geopolitical uncertainty. Rather than relying on formal agreements or institutional continuity, partnerships were often maintained through the persistence and commitment of these individuals.

Participants described this work as labour-intensive and requiring a high degree of personal investment. Champions were often willing to take on tasks beyond formal role expectations in order to maintain relationships and advance partnership goals. As Judy (a GN participant) explained, “Basically, Carolyn [another GN partner] and I will do anything and everything – whether it’s washing toilets or shaking hands with the ministers, that’s part of our job.” This willingness to engage in both visible and invisible labour underscores the extent to which partnerships depended on individuals who were deeply committed to the collaboration.

This relational commitment also enabled partnerships to persist through uncertainty. Participants described how, in the face of shifting political landscapes, funding disruptions, and institutional challenges, it was the dedication of these individuals that allowed collaboration to continue. Beverly (a GN participant) reflected on this persistence, stating, “Unfortunately, I’m the only [GN] partner left (…) but I hung in there because I truly believe in the project (…) these projects are long and labour-intensive (…) what I could do quickly at my own university takes years in the partnership process.” These accounts demonstrate that champions functioned as key relational anchors within partnerships, enabling continuity even when structural supports were unstable or absent.

### Shared values and the enactment of an anticolonial ethos

In addition to the role of champions, participants emphasized that partnerships were oriented around shared values that made up an anticolonial ethos which shaped how collaboration was enacted. These values included humility, mutual respect, reciprocity, and a commitment to equity and were described as guiding principles for how partners engaged with one another and made decisions within the partnership.

Participants described these values as being enacted through everyday relational practices rather than formalized through institutional agreements. Winnie (a GS participant) highlighted the importance of developing relationships grounded in mutual understanding prior to collaboration, noting, “Before we even agree that this should start as a collaboration, people need to get to know each other and understand the way they do things (…) You study each other and ask yourself, are these not only friends but a team where we could work together?” Similarly, Tessa (a GS participant) described the importance of humility in shaping partnership engagement, explaining that ethical collaboration required “the humility to say, ‘how can I help you?’ instead of ‘I want to come help you in my way.’”

These practices reflect what can be understood as an anticolonial ethos within partnership work; an orientation that resists hierarchical and deficit-based approaches by prioritizing listening, responsiveness to context, and equitable sharing of responsibility and benefits. Participants described how this ethos shaped not only interpersonal relationships but also how decisions were made and whose priorities were centered. These relational commitments were described by both GN and GS participants, although GS participants more frequently emphasized the importance of being heard, respected, and able to shape partnership priorities.

These findings illustrate that partnerships were sustained not only through the presence of committed individuals, but through shared values that oriented how relational labour was enacted over time.

### Processes: Negotiating partnership work

While people provided the relational foundation for partnership work, participants emphasized how partnership processes shaped what became possible within collaboration. Participants emphasized that how partnerships unfolded – the processes through which decisions were made, priorities were negotiated, and work was carried out – was central to shaping collaboration. Processes were not experienced as neutral or technical pathways toward predefined outcomes, but as forms of ongoing work that required negotiation, adaptation, and continued engagement. In this way, processes shaped not only what partnerships produced, but how collaboration was enacted within specific institutional, contextual, and geopolitical constraints.

### Processes as negotiated, collaborative work

Participants described partnership processes as requiring continuous negotiation of roles, expectations, and priorities rather than the implementation of fixed or predetermined plans. This work often involved navigating differing institutional pressures, professional norms, changes in demands and capacities, requiring flexibility and ongoing recalibration over time. Michelle (a GS participant) described how this negotiation was iterative as partnerships developed saying, “I think for us as time has evolved, we needed to establish what our role was and what we needed from them (…) it became more and more important for us to kind of stand up and start to put barriers that told them, ‘This is the point that we need to stay at.’” Such moments of boundary-setting were not isolated occurrences but an ongoing dance that required partners to continually reassess what was feasible and appropriate within their specific local context and given timeframe.

Participants also emphasized that collaboration required engaging with different ways of knowing and doing, particularly when institutional norms or professional practices differed across contexts. Beverly (a GN participant) reflected on how partnership processes challenged assumptions about standardized approaches she was accustomed to: “just because I think every child needs to have pulse oximetry when they come in with asthma, does not mean that they do (…) It’s just how I’ve learned to do it, but there’s more than one way to do things.”

Some participants also questioned conventional indicators of partnership success, noting that standard metrics such as publications, institutional reports, or grant outputs did not fully capture the work involved in supporting and strengthening collaboration. Sophie (a GS participant) noted, “It’s actually sad because, in a way, research is favoured [over teaching partnership work between academic institutions] (…) There might not even be a publication coming out of this. But, personally, I see such value in the teaching and learning and the partnership itself.” Instead of emphasizing end-goals, participants continually emphasized the importance of the processes themselves, including the time, effort, and negotiation required to build and maintain partnerships over time. These insights suggest that the work of collaboration, which is often invisible within traditional evaluative frameworks, was itself a meaningful component of partnership activity.

Together, participant accounts suggest that partnership processes involved ongoing negotiation not only of roles and responsibilities, but of meaning, practice, and knowledge legitimacy itself. In this sense, processes functioned as spaces of collaboration through which partners aligned expectations, navigated difference, and challenged personal and institutional assumptions and norms over time.

### Processes as navigating complexity, context, and constraint

In addition to negotiation, participants consistently described partnership processes as unfolding within complex and often constraining institutional and contextual environments. Rather than viewing partnerships as linear or predictable, participants emphasized that collaboration required navigating multiple, intersecting forms of complexity, including organizational and bureaucratic structures, cultural expectations, and broader systemic conditions. Karl (a GN participant) described this complexity as a defining feature of academic global health partnerships, noting that sustainable partnerships required “moving away from ‘this is right, this is wrong,’ and moving into more pluralistic thinking. It’s complex. It’s complex in the GS. It’s complex here in the GN. Let’s try to understand that complexity more.” This orientation toward pluralism and uncertainty was described as essential for working across contexts and understanding each other in a way that kept partnerships going.

Participants also highlighted how institutional processes shaped and, at times, constrained partnership work. Administrative requirements, approval structures, and bureaucratic timelines were frequently described as influencing how quickly decisions could be made and how work progressed. Opembe (a GS participant) reflected on these realities, explaining that “we have a very slow process in deciding and getting things done (…) the approval of curricula, approval of new faculty and so on. It takes unnecessarily long, but I think that’s how the process goes.” Rather than framing these processes solely as barriers, participants described the need to work within these structures while remaining attentive to local institutional norms and expectations.

Although institutional and bureaucratic structures were generally experienced as constraints that partners needed to navigate rather than transform, participants described a few instances in which partnership arrangements were purposefully adapted to create greater flexibility. For example, Diego (a GS participant) deliberately advocated for broad language in the partnership memorandum of understanding that “left the door open” for additional collaborations to emerge over time, including research projects and invitations for partners to teach at the partnering university. Partners also adapted their approach to navigating institutional approval processes. When Winnie (a GS participant) and Beverly (a GN participant) encountered difficulty gaining support from government, university, and regulatory leaders for a new graduate nursing program, they convened these stakeholders through a conference and presented an assessment demonstrating the need for the program. These examples illustrate that while participants rarely altered the institutional systems within which partnerships operated, they sometimes adapted the structures and processes governing the partnership to accommodate local priorities and changing circumstances. Thus, rather than attempting to change broader bureaucratic structures, participants generally worked within or around them, using flexibility, persistence, and relationship-building to move the partnership forward.Participants also highlighted how broader organizational and political changes could significantly disrupt partnership processes. Ruby (a GN participant) described how leadership turnover created instability within one collaboration saying, “There were just so many changes in leadership and in administrative stuff that there was a bit of a vacuum (…) there was about an 18-month timeframe there where things were at a standstill in the partnership.” Across interviews, participants illustrated how partnership processes were shaped not only by the work of collaboration itself, but by broader institutional and political conditions that could slow, redirect, or temporarily halt progress. In response, participants described adapting partnership goals and expectations to align with shifting realities, rather than adhering to fixed timelines or predefined outcomes.

Taken together, these findings demonstrate that partnership processes were dynamic and contextually situated forms of work. Through ongoing negotiation and adaptation, participants sought to persevere in collaboration while navigating the complexities and constraints inherent to global health partnerships in a changing world.

### Power: Structural, institutional, and interpersonal dynamics

Power was not experienced as a discrete element of partnership, but as a pervasive force shaping what was possible within collaboration. Despite being explicitly asked about power dynamics, participants often described relationships and the work of partnership without explicitly naming power. Participants generally found it easier to discuss power through concrete dimensions such as decision-making, agenda setting, leadership, and the distribution of benefits. This tension between the presence of power structures and its relative absence from explicit discussion surfaced as a defining feature of academic global health partnerships.

### Power as structuring forces in partnership

Participants described partnerships as situated within broader global and institutional structures that reflected enduring inequities between the GN and GS. These inequities were rooted in colonial histories and continue through contemporary forms of global health practice, shaping whose knowledge and experience were privileged, whose expertise was legitimized, and whose priorities were advanced. In this way, partnerships were not neutral spaces of collaboration but were embedded within ongoing neocolonial relations. These dynamics were often expressed through discourse and assumptions about capacity and expertise. Charmaine (a GS participant) described how some GN partners approached collaboration saying, “They really do sometimes think that they are almost God-like in how they’re going to help us.” Similarly, Carolyn (a GN participant) reflected on how assumptions about poverty became conflated with assumptions about intelligence noting, “Because [many of the GS contexts we partner with] are very poor, sometimes GN people make an assumption that the people aren’t smart (…) It’s not true, but that plays into a power differential.” This reflects how deficit-oriented framings of GS contexts can reproduce epistemic hierarchies, positioning GN partners as donors and GS partners as recipients. Such assumptions, even when implicit or well-intentioned, contributed to the marginalization of locally situated knowledge and reinforced unequal relations within partnerships.

Participants also emphasized that access to financial resources played a central role in shaping power relations. Control over funding influenced decision-making authority, partnership priorities, and the overall direction of collaboration. Ruby (GN participant) described how discussions about “who’s funding what” and what each partner could afford financially were intertwined with considerations of benefits and risks for each partner. Other participants from both GN and GS countries noted that those with greater access over financial resources often had disproportionately greater capacity to shape outcomes, even within partnerships committed to equity and decolonization. In this way, power operated not only through discourse, but through material conditions that structured whose voices carried weight within collaboration.

Historical, geopolitical, and racialized contexts further shaped how power was experienced within partnerships. Gabe (GN participant) reflected on how colonial histories influenced interpersonal dynamics: “People immediately assumed I was from [a European country that colonized a specific African country] (…) So, I think history also made people very leery of me. It did take time to kind of convince people that I wasn’t an overtly racist White person.” In contrast, Brian (a GN participant) described how his racial and ethnic identity facilitated trust and access to local knowledge. He explained that, when working in GS countries where populations were predominantly Brown, he was readily included in conversations with local colleagues: “One of the advantages that I have is that we often work in countries that are predominantly Brown and I’m frequently the only Brown person [from GN] on the project. And so, people [in the GS] talk to me.” He described experiencing partnerships as both an insider and outsider, with his ethnic similarity to local partners facilitating access to contextual knowledge and trust. These accounts illustrate how colonial legacies and racialized assumptions shaped trust, perception, and engagement within partnerships, underscoring that power was not only structural but historically and socially situated.

### Power as expressed through relationships and processes

While structural forces shaped the conditions of partnership, power was continually enacted through the people and processes that defined collaboration. Participants described how power was enacted in everyday interactions, often in subtle and indirect ways, shaping how individuals were positioned, how decisions were made, and how partnership activities unfolded. In this way, power was not external to partnership work but constituted through the very relationships and processes that defined collaboration. Several participants described how power operated through assumptions about expertise and authority in practice. In reflecting on clinical decision-making, Brian (a GN participant) noted that “you have the local cardiologist who’s been doing this for 30 years [in GS], but people are deferring to somebody who’s doing it for five [from GN]”, highlighting how authority was often granted to GN-trained professionals despite the substantial local expertise of GS-trained professionals. This illustrates how epistemic power operated within partnerships, reinforcing hierarchies that privileged certain forms of training and knowledge over contextually grounded experience. Participants also described how structural resource asymmetries were enacted through everyday interactions, shaping expectations, roles, and decision-making within partnerships.

While many participant accounts reflected inequitable power relations shaped by colonial legacies and contemporary global health structures, there were also instances in which participants resisted or actively worked to disrupt these dynamics. Participants described moments in which power was recognized, reflected upon, and reoriented in ways that challenged neocolonial assumptions positioning the GN as superior and the GS as inferior. In some instances, GS partners asserted boundaries, redefined roles, and resisted expectations that did not align with local priorities. Participants also described using relationships as a means of reorienting power, working toward mutual respect and recognition of each other’s expertise. These efforts highlight that power, while pervasive, was not experienced as fixed or unidirectional. In reflecting on this fluidity, Nia (a GS participant) emphasized that “nobody has power [all the time] (…) power will fluctuate based on the outcome of the goals of the partnership,” noting that “at a point, I will have the power, at another point, they have the power.” This perspective complicates static understandings of power by highlighting how it is continuously reconfigured through decision-making, context, and the evolving goals of the partnership.

Many participants also demonstrated critical personal reflection in relation to power, acknowledging its presence and, in some cases, intentionally working to address it. Diego (a GS participant) suggested that “being more aware of power dynamics is definitely one way that universities can then be better partners,” emphasizing the importance of attending to “historical relationships, governance mechanisms, [and] best practices” in shaping more equitable collaboration. Such reflections demonstrate that participants were not only navigating power but also engaging in practices that challenged dominant assumptions and worked toward more equitable and contextually grounded forms of partnership.

These findings demonstrate that power permeated academic global health partnerships not only as a source of constraint, but also as a site of ongoing negotiation, reflection, and, at times, resistance. While structural inequities continued to shape what was possible, participants’ accounts highlight the ways in which power was reworked through relationships and processes in pursuit of more equitable partnerships.

## Discussion

### Synthesis of findings

This study examined how academic global health partnerships in health professions education are enacted through the interrelated dynamics of people, processes, and power. Across interviews with experienced GN and GS partners, partnerships were not maintained primarily through formal agreements, institutional structures, or predefined outcomes. Instead, they were maintained through relational labour of champions, enacted through negotiated and contextually situated processes, and shaped by enduring structural and epistemic power asymmetries.

Participants emphasized that partnerships were fundamentally human endeavours, carried out by champions who invested significant emotional and relational effort over time. These individuals acted as anchors during periods of instability, enabling partnerships to persist despite funding disruptions, institutional turnover, and geopolitical uncertainty. The centrality of relational labour in sustaining partnerships aligns with scholarship that conceptualizes global health collaborations as fundamentally relationship-based enterprises rather than mechanisms for transferring resources or expertise [57–59]. Furthermore, equitable partnerships require commitment to decolonial values and actions such as humility, mutual learning, and recognition of local expertise, emphasizing that successful collaborations depend on the quality of relationships among partners rather than the formal structures that connect them [18,60,61].

At the same time, partnerships unfolded through complex processes that required ongoing negotiation of roles, expectations, and priorities. Rather than linear or predictable, partnership work was iterative, shaped by contextual constraints and shifting institutional realities. These findings support calls for greater attention to the governance and operational processes through which partnerships are enacted [28,62,63]. Decolonial partnerships cannot be achieved through goodwill alone but must be embedded within partnership structures through shared decision-making, transparent communication, reciprocal accountability, and meaningful participation in agenda-setting and implementation [59–61,64]. Our findings suggest that such processes are not simple mechanisms but ongoing sites of negotiation that require continuous adaptation to changing contexts.

Power operated across all aspects of partnership work. Structurally, partnerships were embedded within neocolonial systems that privileged GN institutions through access to resources, mobility, and epistemic authority. Power was expressed through both relationships and processes, shaping whose expertise was recognized, how decisions were made, and how collaboration was experienced. Importantly, participants also described moments of reflection and resistance, in which partners challenged dominant assumptions, asserted local priorities, and worked toward more equitable forms of collaboration. These findings are consistent with critiques of global health partnerships that highlight how historical and contemporary power asymmetries inform whose priorities are advanced, whose voices carry authority, and whose knowledge is valued [18,20,61]. These inequities also extend beyond individual partnerships to broader global workforce dynamics. The sustainability challenges experienced by many GS health systems are shaped not only by local constraints but also by international patterns of health worker migration (i.e., brain drain) and unequal investments in health professions education, through which many GN countries continue to benefit from expertise developed elsewhere [1,3]. Furthermore, the findings of this study resonate with recent discussions of epistemic injustice in global health, arguing that decolonizing partnerships requires not only redistributing resources and decision-making authority but also recognizing diverse forms of knowledge and challenging the privileging of Western epistemologies [19,56,64]. The relative absence of explicit discussion using the word “power” is also notable given that questions addressing the concept were asked during the interviews. Participants’ greater ease discussing leadership, resources, or equity suggests that power may be implicitly understood in its operationalization rather than discussed overtly as an explicit, political dimension of capacity-building and knowledge production in global health partnerships. The results of this study demonstrate that partnerships are supported and maintained not only through technical inputs, but through the ongoing negotiation of relationships, processes, and power. This perspective challenges dominant models of global health collaboration that prioritize measurable outputs over relational and contextual dimensions of partnership work [4,8]. Rather than viewing partnership success as the product of formal structures or predefined outcomes, our findings suggest that success may, in fact, be found in the interaction of relational commitments, negotiated processes, and ongoing efforts to address power asymmetries.

### Reframing global health workforce development: From brain drain to brain sustain

The findings of this study offer an opportunity to reframe how global health partnerships contribute to workforce development, which is an essential component for healthcare sustainability worldwide [1,3]. Traditionally, discussions of workforce sustainability in global health have been influenced by the concept of brain drain, which highlights the migration of health professionals from GS to GN and its impact on national health systems that workers leave behind [2,29,31]. While this framing has been important for identifying structural inequities in global labour markets, it often conceptualizes the problem at the level of individual mobility, with less attention to the broader relational, institutional, and structural conditions that shape workforce development internationally [3].

Participants in this study described a more complex reality. Successful partnerships were often oriented toward strengthening health professions education systems, supporting faculty development, and building institutional capacity within GS contexts. These efforts align with existing literature that emphasizes the importance of long-term, institution-to-institution partnerships for strengthening national health workforces [6,8,9]. However, participants also expressed ambivalence about whether such partnerships could fully achieve these goals within existing global structures, particularly when power asymmetries and institutional incentive systems continued to privilege GN priorities.

The findings of this study also illustrate both the promise and the fragility of a decolonial approach. Participants described partnerships that were actively working toward more equitable and contextually grounded forms of collaboration and knowledge exchange and knowledge production, often through acts of resistance to neocolonial power as well as relational commitment. At the same time, these efforts were constrained by broader structural forces, including cancelled funding mechanisms, academic evaluation systems, and enduring geopolitical inequities. These findings suggest that brain sustain operates at both a practical and aspirational level. It is grounded in strategies that participants had already enacted to sustain partnerships and advance locally grounded health professions education despite structural constraints, while also offering an aspirational orientation for academic institutions and partnership actors. We do not suggest that individual partnerships can independently alter the broader geopolitical, economic, or institutional structures within which global health partnerships operate. Rather, brain sustain draws attention to the forms of agency available within these constraints and to how the lessons from participants’ experiences may inform partnership practices in the current context.

Understanding health workforce development through an augmented lens highlights a central paradox of contemporary global health partnerships: even as partners seek to move beyond extractivist models and invest in meaningful, nationally or locally grounded capacity building, they do so within systems that continue to privilege Western knowledge systems and academic productivism. Addressing this paradox requires not only new concepts but also continued attention to how partnerships are enacted in practice.

In response to this paradox, we propose the concept of brain sustain, as defined in the introduction, as a way of rethinking workforce development in the context of maintaining universal healthcare systems as a moral, political, and economic imperative for global health that is a collective responsibility [1,28]. Brain sustain is not a technical solution but a decolonial orientation to educational partnership work that responds to calls to centre epistemic justice, the redistribution of authority, and the recognition of diverse ways of knowing [15,20,23,38]. This orientation also recognizes that responsibility for workforce sustainability is shared collectively and globally. Brain sustain, therefore, draws attention not only to strengthening educational capacity within GS contexts but also to the structural conditions through which GN countries and institutions benefit from internationally trained health professionals and contribute to persistent workforce inequities [1,3]. Rather than focusing on the movement or loss of individual health professionals from the GS to the GN (i.e., brain drain), brain sustain directs attention to the broader educational, institutional, and professional environments that enable locally grounded and sustainable health professions education systems to flourish. This includes strengthening existing national training programs, supporting capacity development within public universities, and valuing the contributions of GS champions within their own institutions. We therefore conceptualize “brain sustain partnerships” as equitable collaborations that foster knowledge exchange, knowledge generation, educational initiatives and programs, and teaching capacity strengthening in ways that reinforce local workforce capacity and support publicly funded healthcare systems. Although no single initiative can achieve these goals alone, partnerships can be intentionally oriented toward creating and supporting the conditions that make brain sustain a viable possibility. Our findings suggest that such conditions include recognizing the relational labour invested by champions, engaging with partnership processes as complex and iterative rather than linear, and addressing power as a pervasive force that shapes decision-making, expertise, and opportunities for collaboration. Brain sustain should be viewed as both a partnership aspiration and a structural challenge. Although individual collaborations cannot resolve the political and economic forces that contribute to workforce inequities, they operate within (and are affected by) global systems of labour mobility, educational investment, and resource distribution. Advancing brain sustain therefore requires both equitable partnership practices and broader commitments to workforce sustainability, particularly from GN institutions and countries that benefit from the migration of health professionals trained elsewhere [1,3]. By intentionally supporting locally grounded health professions education, workforce capacity building, and equitable systems of knowledge exchange, brain sustain partnerships may help shift global health collaboration away from technocratic programs and extractivist models and toward approaches that contribute to the long-term sustainability of health workforces and healthcare systems in GS countries.

### Limitations

This study has a few notable limitations. First, participants represented a self-selected group of individuals with extensive experience in global health partnerships; as such, their perspectives may not reflect the full range of experiences across academic global health partnerships between the GN and GS. Second, it is also possible that participants selectively shared their accounts, particularly given the relational and professional sensitivities of partnership work. Power differentials between the interviewer and participants (especially those based in GS) may have shaped the depth and nature of responses, with some interviews yielding more concise accounts despite the use of dialogical techniques. Third, while this study was informed by postcolonial theory, the focus on individual experiences means that broader structural comparisons across countries and systems were not fully captured. Finally, as the first author, RKE, is a white Canadian novice researcher working within Western academic conventions, perhaps some aspects of participants’ perspectives were interpreted through dominant epistemic frameworks, despite efforts to engage reflexively and incorporate decolonizing approaches.

### Implications for research, education, and practice

Future global health partnership research must prioritize decolonial approaches that centre equitable collaboration between GN and GS scholars as well as meaningfully incorporate GS perspectives and expertise. Future research should also focus on the impact of global geopolitical shifts on academic global health partnerships. For health professions education and global health education programs, the findings underscore the need to prepare learners and faculty to engage critically with global health partnerships. This includes teaching reflective practice and theories of power. For universities, funders, and global health institutions, these findings point to the need for recognizing and resourcing the relational and process-oriented labour that sustains partnerships, revisiting how success is evaluated, and redistributing decision-making authority are critical for aligning partnership practices with commitments to equity, decolonization, and the development and sustainability of national healthcare systems. Without such shifts, partnerships risk reproducing the inequities they intend to dismantle.

## Conclusion

This study offers both empirical and conceptual contributions to understanding academic global health partnerships. Empirically, it demonstrates that partnerships are not sustained primarily through formal arrangements, but through the interrelated dynamics of people, processes, and power. By foregrounding relational labour, negotiated processes, and the pervasive influence of structural and epistemic power, the findings challenge conventional models of collaboration that prioritize measurable outputs over the conditions that make partnership possible. Conceptually, introducing the concept of brain sustain reframes workforce development in global health as a collective, relational, and political endeavour, rather than an issue of individual mobility alone. Together, these contributions highlight the need to move beyond technical approaches to partnership toward more critically reflexive, equity-oriented, and contextually meaningful practices in global health.

## Supporting information

S1 Appendix Interview Guide. (DOCX)

## References

[1] McKee M. Global sustainable healthcare. Medicine (Baltimore). 2018;46(7):383–7. doi: 10.1016/j.mpmed.2018.04.008

[2] Dodani S, LaPorte RE. Brain drain from developing countries: how can brain drain be converted into wisdom gain? J R Soc Med. 2005;98(11):487–91. 10.1258/jrsm.98.11.487 PMC1275994 16260795

[3] McPake B, Dayal P, Zimmermann J, Williams GA. How can countries respond to the health and care workforce crisis? Insights from international evidence. Int J Health Plann Manage. 2024;39(3):879–87. 10.1002/hpm.3766 38278780

[4] Citrin D, Mehanni S, Acharya B, Wong L, Nirola I, Sherchan R, et al. Power, potential, and pitfalls in global health academic partnerships: review and reflections on an approach in Nepal. Glob Health Action. 2017;10(1):1367161. doi: 10.1080/16549716.2017.1367161 28914185 PMC5645653

[5] Farmer PE, Rhatigan JJ. Embracing Medical Education’s Global Mission. Acad Med. 2016;91(12):1592–4. doi: 10.1097/ACM.0000000000001433 27749305

[6] Frenk J, Chen L, Bhutta ZA, Cohen J, Crisp N, Evans T, et al. Health professionals for a new century: transforming education to strengthen health systems in an interdependent world. Lancet. 2010;376(9756):1923–58. doi: 10.1016/S0140-6736(10)61854-5 21112623

[7] Larson CP, Plamondon KM, Dubent L, Bicaba F, Bicaba A, Minh TH, et al. The Equity Tool for Valuing Global Health Partnerships. Glob Health Sci Pract. 2022;10(2):e2100316. doi: 10.9745/GHSP-D-21-00316 35487557 PMC9053142

[8] Rabin TL, Mayanja-Kizza H, Rastegar A. Medical Education Capacity-Building Partnerships for Health Care Systems Development. AMA J Ethics. 2016;18(7):710–7. doi: 10.1001/journalofethics.2016.18.7.medu2-1607 27437821

[9] Spies LA, Garner SL, Faucher MA, Hastings-Tolsma M, Riley C, Millenbruch J, et al. A model for upscaling global partnerships and building nurse and midwifery capacity. Int Nurs Rev. 2017;64(3):331–44. doi: 10.1111/inr.12349 28261789

[10] Adam M, LaBeaud D, Mbewu N, Gates J, Waechter R, Borbor-Cordova MJ, et al. Protecting global health partnerships in the era of destructive nationalism. PLOS Glob Public Health. 2025;5(4):e0004428. doi: 10.1371/journal.pgph.0004428 40198681 PMC11977972

[11] Afriyie EK, Mbwoge RN, Koray MH. Decolonizing global health in an age of fragmentation: reimagining equity for universal health coverage. Health Policy Plan. 2025;41(2):336–9. doi: 10.1093/heapol/czaf109 41378772 PMC12906760

[12] Gostin LO. The Trump presidency: Cascading global shocks on global health. PLOS Glob Public Health. 2025;5(11):e0005385. doi: 10.1371/journal.pgph.0005385 41296701 PMC12654930

[13] Gostin LO, Meier BM, Pace L. A World Without WHO-A Crossroads for US Global Health Leadership. JAMA. 2025;333(12):1028–9. doi: 10.1001/jama.2024.28827 39823211

[14] Abimbola S. The foreign gaze: authorship in academic global health. BMJ Glob Health. 2019;4(5):e002068. doi: 10.1136/bmjgh-2019-002068 31750005 PMC6830280

[15] Bhakuni H, Abimbola S. Epistemic injustice in academic global health. Lancet Glob Health. 2021;9(10):e1465–e70. doi: 10.1016/S2214-109X(21)00301-6 34384536

[16] Abimbola S, Pai M. Will global health survive its decolonisation? Lancet. 2020;396(10263):1627–8. doi: 10.1016/S0140-6736(20)32417-X 33220735

[17] Binagwaho A, Allotey P, Sangano E, Ekström AM, Martin K. A call to action to reform academic global health partnerships. BMJ. 2021;375:n2658. doi: 10.1136/bmj.n2658 34725093

[18] Garba DL, Stankey MC, Jayaram A, Hedt-Gauthier BL. How Do We Decolonize Global Health in Medical Education? Ann Glob Health. 2021;87(1):29. doi: 10.5334/aogh.3220 33816134 PMC7996454

[19] Affun-Adegbulu C, Adegbulu O. Decolonising Global (Public) Health: from Western universalism to Global pluriversalities. BMJ Glob Health. 2020;5(8):e002947. doi: 10.1136/bmjgh-2020-002947 32819916 PMC7443258

[20] Eichbaum QG, Adams LV, Evert J, Ho M-J, Semali IA, van Schalkwyk SC. Decolonizing Global Health Education: Rethinking Institutional Partnerships and Approaches. Acad Med. 2021;96(3):329–35. doi: 10.1097/ACM.0000000000003473 32349015

[21] Kwete X, Tang K, Chen L, Ren R, Chen Q, Wu Z, et al. Decolonizing global health: what should be the target of this movement and where does it lead us? Global Health Res Policy. 2022;7(1):3. 10.1186/s41256-022-00237-3 PMC8784247 35067229

[22] Khan M, Abimbola S, Aloudat T, Capobianco E, Hawkes S, Rahman-Shepherd A. Decolonising global health in 2021: a roadmap to move from rhetoric to reform. BMJ Glob Health. 2021;6(3):e005604. doi: 10.1136/bmjgh-2021-005604 33758016 PMC7993212

[23] Hafez S, Clarke A, Richter K, Lokot M, Rivas A-M, Singh NS. Dismantling colonial legacies: Decolonising research and teaching at the Health in Humanitarian Crises Centre, London school of hygiene and tropical medicine. PLOS Glob Public Health. 2025;5(7):e0004833. doi: 10.1371/journal.pgph.0004833 40700352 PMC12286357

[24] McCoy D, Kapilashrami A, Kumar R, Rhule E, Khosla R. Developing an agenda for the decolonization of global health. Bull World Health Organ. 2024;102(2):130–6. doi: 10.2471/BLT.23.289949 38313156 PMC10835633

[25] Santos BS. The university at a crossroads. In: Grosfoguel R, Hernández R, Velásquez ER, editors. Decolonizing the Westernized University: Interventions in Philosophy of Education from Within and Without. Lanham (MD): Lexington Books; 2016.

[26] Santos BS. The end of the cognitive empire: The coming of age of epistemologies of the South. Durham (NC): Duke University Press; 2018.

[27] Finkel ML, Temmermann M, Suleman F, Barry M, Salm M, Bingawaho A, et al. What Do Global Health Practitioners Think about Decolonizing Global Health? Ann Glob Health. 2022;88(1):61. doi: 10.5334/aogh.3714 35974980 PMC9336780

[28] Kulesa J, Brantuo NA. Barriers to decolonising educational partnerships in global health. BMJ Glob Health. 2021;6(11):e006964. doi: 10.1136/bmjgh-2021-006964 34789513 PMC8601064

[29] Arfeen Z, Nash E, Rashid MA. Reliance on migrant healthcare workers in the United Kingdom: A critical discourse analysis. PLOS Glob Public Health. 2025;5(9):e0005141. doi: 10.1371/journal.pgph.0005141 40911622 PMC12412976

[30] Mills EJ, Schabas WA, Volmink J, Walker R, Ford N, Katabira E, et al. Should active recruitment of health workers from sub-Saharan Africa be viewed as a crime? Lancet. 2008;371(9613):685–8. doi: 10.1016/S0140-6736(08)60308-6 18295027

[31] Vega-Muñoz A, Gónzalez-Gómez-del-Miño P, Espinosa-Cristia JF. Recognizing New Trends in Brain Drain Studies in the Framework of Global Sustainability. Sustainability. 2021;13(6):3195. doi: 10.3390/su13063195

[32] Bickford D. Postcolonial theory, nursing knowledge, and the development of emancipatory knowing. ANS Adv Nurs Sci. 2014;37(3):213–23. doi: 10.1097/ANS.0000000000000033 25102212

[33] Chilisa B, Phatshwane K. Qualitative Research within a Postcolonial Indigenous Paradigm. In: Flick U, editor. The SAGE Handbook of Qualitative Research Design. Thousand Oaks (CA): SAGE Publications Ltd.; 2022.

[34] Demir I. How and Why Should We Decolonize Global Health Education and Research? Ann Glob Health. 2022;88(1):30. doi: 10.5334/aogh.3787 35646615 PMC9122006

[35] Reimer-Kirkham S, Anderson JM. Postcolonial nursing scholarship: from epistemology to method. ANS Adv Nurs Sci. 2002;25(1):1–17. doi: 10.1097/00012272-200209000-00004 12889574

[36] Coombs S, Potts M, Whitehead J. International educational development and learning through sustainable partnerships: Living global citizenship. Basingstoke (UK): Palgrave Macmillan; 2014.

[37] Santos BS. Another knowledge is possible: beyond northern epistemologies. London: Verso; 2008.

[38] Faerron Guzmán CA, Rowthorn V. Introduction to Special Collection on Decolonizing Education in Global Health. Ann Glob Health. 2022;88(1):38. doi: 10.5334/aogh.3756 35651971 PMC9138903

[39] Racine L, Perron A. Unmasking the predicament of cultural voyeurism: a postcolonial analysis of international nursing placements. Nurs Inq. 2012;19(3):190–201. 10.1111/j.1440-1800.2011.00555.x 22882504

[40] Kahlke RM. Generic Qualitative Approaches: Pitfalls and Benefits of Methodological Mixology. Int J Qual Methods. 2014;13(1):37–52. doi: 10.1177/160940691401300119

[41] Caelli K, Ray L, Mill J. ‘Clear as Mud’: Toward Greater Clarity in Generic Qualitative Research. Int J Qual Methods. 2003;2(2):1–24. doi: 10.1177/160940690300200201

[42] Gastaldo D. Theoretical congruence and rigour in qualitative research [Video file]. Centre for Critical Qualitative Health Research; 2018.

[43] Merriam SB, Tisdell EJ. Qualitative research: A guide to design and implementation. 4th ed. San Francisco (CA): Jossey-Bass. 2016.

[44] Roulston K, Halpin S. Designing Qualitative Research Using Interview Data. In: Flick U, editor. The SAGE Handbook of Qualitative Research Design. Thousand Oaks (CA): SAGE Publications Ltd.; 2022.

[45] Smith LT. Decolonizing methodologies: Research and Indigenous peoples. 2nd ed. London: Zed Books; 2012.

[46] Cockell J, McArthur-Blair J. Appreciative inquiry in higher education: A transformative force. San Francisco (CA): Jossey-Bass; 2012.

[47] Norum KE. Appreciative inquiry. In: Given LM, editor. The SAGE encyclopedia of qualitative research methods. Thousand Oaks (CA): SAGE Publications; 2008.

[48] Malterud K, Siersma VD, Guassora AD. Sample Size in Qualitative Interview Studies: Guided by Information Power. Qual Health Res. 2016;26(13):1753–60. doi: 10.1177/1049732315617444 26613970

[49] Tracy SJ. Qualitative quality: Eight “big-tent” criteria for excellent qualitative research. Qual Inq. 2010;16(10):837–51.

[50] Creswell JW. Research design: Qualitative, quantitative, and mixed methods approaches. 2nd edition. Thousand Oaks (CA): SAGE Publications; 2003.

[51] Kennedy BL, Thornberg R. Deduction, Induction, and Abduction. In: Flick U, editor. The SAGE Handbook of Qualitative Data Collection. London: SAGE Publications Ltd.; 2018. p. 49–64.

[52] Patton MQ. Qualitative research and evaluation methods. 4th edition. Thousand Oaks (CA): SAGE Publications; 2015.

[53] Thornberg R. Abduction as a Guiding Principle in Qualitative Research Design. In: Flick U, editor. The SAGE Handbook of Qualitative Research Design. Thousand Oaks (CA): SAGE Publications Ltd; 2022.

[54] Enns RK. Decolonizing global health partnerships: exploring internationalpartnerships in health professions education through a postcolonial lens [PhD dissertation]. Toronto (ON): University of Toronto; 2023.

[55] Olmos-Vega FM, Stalmeijer RE, Varpio L, Kahlke R. A practical guide to reflexivity in qualitative research: AMEE Guide No. 149. Med Teach. 2023;45(3):241–51. 10.1080/0142159X.2022.2057287 35389310

[56] Plamondon KM, Brisbois B, Dubent L, Larson CP. Assessing how global health partnerships function: an equity-informed critical interpretive synthesis. Glob Health. 2021;17(1):73. doi: 10.1186/s12992-021-00726-z 34215301 PMC8254362

[57] Lelli Chiesa P, Osman OTM, Aloi A, Andriani M, Benigni A, Catucci C, et al. Improving standard of pediatric surgical care in a low resource setting: the key role of academic partnership. Ital J Pediatr. 2020;46(1):80. doi: 10.1186/s13052-020-00827-2 32517726 PMC7285579

[58] Virani S, Handuleh JIM, Pereira-Sanchez V, Wolde-Giorgis DF. Teaching psychiatry in a low-income country during the COVID-19 pandemic: A hybrid collaborative psychiatry course. Asia Pac Psychiatry. 2021;13(4):e12503. doi: 10.1111/appy.12503 34967115

[59] Wondimagegn D, Pain C, Baheretibeb Y, Hodges B, Wakma M, Rose M. Toronto Addis Ababa Academic Collaboration: A relational partnership model for building educational capacity between a high- and low-income university. Acad Med. 2018;93(12):1795–801. 10.1097/ACM.0000000000002352 PMC6282678 29995668

[60] Amisi JA, Cuba-Fuentes MS, Johnston EM, Makwero M, Prasad S, Ras T, et al. A pragmatic approach to equitable global health partnerships in academic health sciences. BMJ Glob Health. 2023;8(5):e011522. doi: 10.1136/bmjgh-2022-011522 37230545 PMC10230872

[61] Whitehead C, Wondimagegn D, Baheretibeb Y, Hodges B. The International Partner as Invited Guest: Beyond Colonial and Import-Export Models of Medical Education. Acad Med. 2018;93(12):1760–3. doi: 10.1097/ACM.0000000000002268 29727317

[62] Advocacy for Global Health Partnerships. Brocher Declaration. Available from: https://www.ghpartnerships.org/brocher 10.5334/aogh.3577 PMC9122001 35646612

[63] McGibbon E, Mulaudzi FM, Didham P, Barton S, Sochan A. Toward decolonizing nursing: the colonization of nursing and strategies for increasing the counter-narrative. Nurs Inq. 2014;21(3):179–91. doi: 10.1111/nin.12042 23837570

[64] Wondimagegn D, Whitehead CR. Addressing epistemic injustice (and ongoing effects of colonisation) through the Ethiopian intellectual tradition of Qiné. BMJ Glob Health. 2025;10(7):e019230. doi: 10.1136/bmjgh-2025-019230 40664432 PMC12265799

