# Supplementary material for: Brain sustain partnerships: Equity-orientation and decolonization theory to promote Global North-Global South collaborations in health professions education
Source: PLOS Glob Public Health. 2026 Nov 11;6(11):e0007630. doi: 10.1371/journal.pgph.0007630 (PMC13754107; doi:10.1371/journal.pgph.0007630)
Supplement: Supplementary file 1 [file pgph.0007630.s001.docx]

Interview Guide

1. Could you tell me about how the partnership came to be? (Tell me the story of this partnership)

Probes:

a) How was the partnership initiated?
b) Why was it created?
c) What disciplines were involved?
d) What was your role in the partnership?
2. Could you tell about the best moments in the partnership? *(incorporating the principle of story telling) (this can touch on both process and outcomes)*

Probes:

a) In what ways was the partnership successful?
b) How do you define a successful partnership?
c) One story about process and one story about outcomes.
3. Could you tell me a story about the challenges you faced in your partnership? *(incorporating the principle of story telling) (this can touch on both process and outcomes)*

Probes:

a) Why were those things challenges (in your opinion)?
b) What do you think are the major challenges for partnerships like these or international partnerships?
c) One story about process and one story about outcomes.
4. When I read papers about partnerships, they rarely talk about power dynamics. In your experience, what were the main power dynamics that shaped your partnership? And how were power relations/issues talked about amongst the group/partnership members?

Probes:

a) Talk about agenda setting and decision making
b) What determines who has power in partnerships
5. If you were to start this partnership over again, what would the ideal partnership look like? *(Ideal position question)*

Consider:

a) how were decisions made (relates to power); leadership roles and how leadership was shared (relates to power);
b) operational features (time constraints/on-the-ground presence; clearly aligned goals; participatory/long-term partnership)
c) philosophical principles/values (reciprocity; flexibility/adaptability; how to build mutual trust/confidence/respect)
d) what was achieved (outcome content);

6. What were the benefit from the partnership to your country? What were the benefits to the other country?

Probe:

a) Were the benefits equitable?

7. How did your institution/university benefit from the partnership?

Probe:

a) Were the benefits equitable?
b) Describe the outcomes of the partnership.

8. How did you personally benefit from the partnership?
9. What is needed in order to decolonize global health partnerships? How can we work towards decolonizing global health partnerships?
   a) *(Note: this question was only asked to the last four participants recruited after analyzing the previous participant transcripts)*
10. Insert the question that the participant wants to be asked as well as the question that the participant wants the partner from the country from the opposite income status to be asked
11. Why did you choose to be involved in this study as a participant?
